# Seismic Performance of Reinforced Concrete Columns Retrofitted with Hybrid Concrete Jackets Subjected to Combined Loadings

**DOI:** 10.3390/ma15186213

**Published:** 2022-09-07

**Authors:** Min Sook Kim, Young Hak Lee

**Affiliations:** Department of Architectural Engineering, Kyung Hee University, Deogyeong-Daero 1732, Yongin 17104, Korea

**Keywords:** seismic retrofitting, jacketing, steel grid reinforcement, combined loading

## Abstract

In the existing reinforced concrete columns where they are insufficient seismic details, critical failure mode such as shear failure can be observed under seismic loads. One strategy for the retrofitting of existing concrete columns is to use concrete jacketing. Concrete jacketing consists of a new concrete layer with longitudinal and transverse reinforcements, and can improve seismic resistance capacity. In this paper, a detail of concrete jacket that can be expected for easy construction and improved adhesion performance of longitudinal and transverse reinforcement was proposed. Additionally, a combined cyclic loading test was conducted to consider the seismic load with multiaxial characteristics. The concrete jacket details utilize three components: Steel Grid Reinforcement (SGR), Steel Wire Mesh (SWM), and Steel Fiber Non-Shrinkage Mortar (SFNM). One RC column with non-seismic details and two jacketed RC columns were fabricated to demonstrate the construction efficiencies and structural capacities of the jacketed columns. Two details of jacketed section were considered as variables. It was observed that the specimens retrofitted with concrete jacket resisted torsional load more than the un-retrofitted specimen in terms of crack and failure mode. The experimental results showed that the maximum load of retrofitted specimens was increased by more than 8 times compared to the un-retrofitted specimen, regardless of the jacket details. Newly designed concrete jacket effectively increased the strength. Compared with the un-retrofitted column, the columns retrofitted with the proposed details achieved significant increase in initial stiffness and energy dissipation.

## 1. Introduction

Non-seismically designed reinforced concrete (RC) columns need to be repaired or retrofitted because they do not possess adequate lateral strength and ductility. One typical retrofitting method, concrete jacketing, is to enlarge the cross section of the existing RC column with a new concrete layer, including longitudinal and transverse reinforcements. This method is known as a useful technique to improve the seismic performance of the column in terms of its axial strength, flexural strength, and ductility. In retrofitting RC columns, the usual practice consists of first assembling a jacketed reinforcement composed of longitudinal and transverse reinforcements, arranging the formwork, and then placing the concrete. To achieve the purpose of the retrofitting method, the treatment of the surface of the old columns must be carefully handled because the composite action between the old column and the new concrete layer can improve the structural performance. It is costly and time consuming [1,2,3]. Julio et al. [1,2] analyzed the influence of interface treatment on the seismic performance of concrete columns retrofitted with concrete jacket. They conducted an experiment on seven columns subjected to monotonic and cyclic loading. The roughness of the interface surface, the use of a bonding agent, the added concrete mixture, and application, and steel connectors were considered as variables. The study reveled that the sand blasting is the best roughness treatment and use of epoxy resins does not improve the interface strength. Additionally, high strength concrete increases the interface strength, and use of steel connectors does not significantly increase the interface bonding stress. Vandoros and Dritsos [4] investigated the effect of the construction details of concrete jackets. Three column specimens with the welding jacket stirrup end together, and placing dowels, along with placing bent down steel connectors, were tested. Experimental results showed that the structural performance was improved even when the jacket is constructed with no treatment at the interface. The bent down steel connectors have been proven to increase the energy dissipation capacity. The separation of the jacket from the original column was clearly observed in the case where there was no treatment or other connection.

The arrangement, amount, and mechanical properties of steel reinforcement in the jacketed section are important parameters that impact the effectiveness of its load-carrying capacity and ductility. Sun et al. [5] fabricated five RC column specimens with longitudinal rebar and 90° hoop-like conventional concrete jacketing and Steel Wire Mesh (SWM) for the secondary reinforcement. The amount of longitudinal reinforcement was a variable of the cyclic tests. As a result, the SWM improved the load-carrying capacity, deformation capacity, and energy dissipation with an increasing reinforcement ratio. Tayeh et al. [6] fabricated eleven beam specimens with three different bonding mechanisms; dowels, roughening, and expansion bolts to investigate the flexural behavior of the jacketed beams strengthened with SWM. They also suggested simplified a structural design for predicting the flexural strength and deflection on the basis of flexural theory, verifying that the experimental results and theoretical analysis are similar. Yang et al. [7] developed a new reinforcement detail for concrete jacketing. This new reinforcement uses V-ties as an alternative approach to the arrangement of supplementary crossties in the RC columns. The length of the leg of the V-tie into the core concrete and the amount of transverse reinforcement were the specimen variables. As a result, V-ties can delay the buckling of the longitudinal bars in the jacketed section by confining the core concrete, including existing columns.

For the concrete jacketing to achieve the purpose of retrofitting, it is necessary to secure the confinement and load-carrying capacity. This depends on the bonding between the old column and the new concrete layer and the details of the jacketed section. The interface treatment to ensure bonding, installation of additional connectors, and welding of rebars to improve confinement effect have been proposed. Concrete jacket is a valuable technique in terms of cost and efficiency. Therefore, this study focused on concrete jacket details that ensured bonding without interface treatment, improved seismic performance, and easy construction. The simple combination of steel wire mesh and steel grid reinforcement allows for easy construction, as it can help practitioners in the site easily apply. 

The proposed details consist of two parts: steel wire mesh (SWM), which can replace interface treatment, and steel grid reinforcement (SGR), which can replace longitudinal and transverse reinforcement. The combination of these two parts can simplify the interface treatment process and the placing or welding reinforcement in the new concrete layer. It is also intended to be advantageous for crack control by pouring steel fiber non-shrinkage mortar (SFNM). In order to evaluate the structural performance of RC columns retrofitted with the proposed details of concrete jacket, a cyclic loading test was conducted under combined loading from a compression, including a bending and torsion moment. This is to consider the seismic excitations due to the multi-directional characteristics of earthquakes. 

## 2. Experimental Program

### 2.1. Proposed Details of the Concrete Jacket

**Figure 1:**
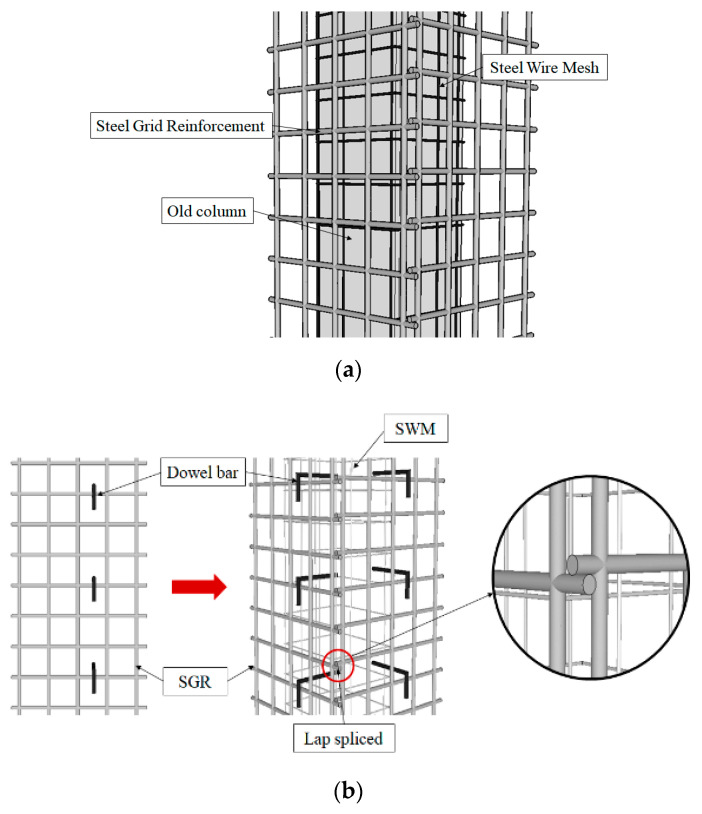

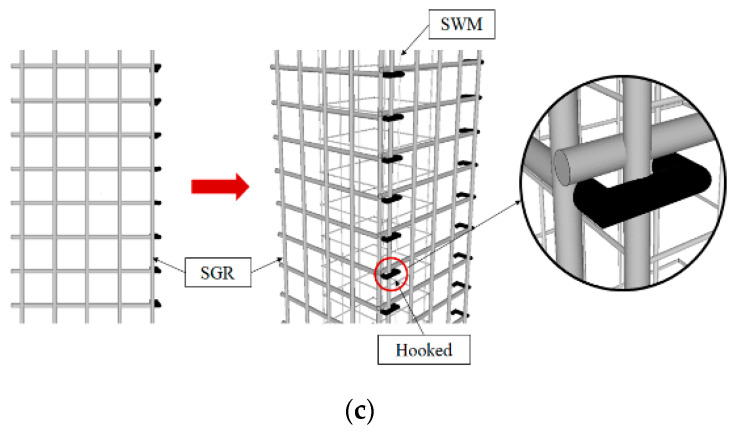
Proposed details of the concrete jackets: (**a**) A combination of SWM and SGR; (**b**) Type 1; (**c**) Type 2.

Two different strengthening details are proposed as alternative methods to the existing concrete jacket. Both proposed reinforcement details of the concrete jacket consist of a combination of steel wire mesh (SWM) and steel grid reinforcement (SGR), as shown in Figure 1a. The SWM is made by welding wires of diameters of 10 mm or 13 mm in the form of a net that can wrap the columns. By applying the SWM, the composite action between the old column and the concrete jacket can be achieved without the use of bonding agents or surface preparation of the old column surface. Using this method, prefabricated SGR can be installed instead of placing longitudinal and transverse reinforcement around the old column. Using SGR, a vertically arranged bar can replace the role of the main bar, and a transversely arranged bar can replace the role of the stirrups. SGRs are introduced for fast and easy reinforcement placing because they can reduce the on-site work for reinforcement placing. In addition, steel fiber non-shrinkage mortar (SFNM) is used to control cracks and improve bond performance. The proposed concrete jacket is constructed in four steps. First, SWM is placed close to the old column surface. Second, considering the concrete cover, the SGR is placed on the four sides of the old column. The SGR is placed using pre-installed holes in the foundation or slab. Third, the old column-SMW-SGR is connected with a dowel bar or hook. Fourth, the formwork is installed and the SFNM is placed. 

Two different methods are proposed to install a combination of SWM and SGR. Type 1 makes a hole in the old column and inserts a dowel bar to fix the combination of SWM and SGR. Type 2 inserts a hook that couples the reinforcing bars placed transversely in the SGR instead of dowel bars. Figure 1b,c show the difference between Type 1 and Type 2 reinforcement. 

### 2.2. Specimen Details

**Figure 2:**
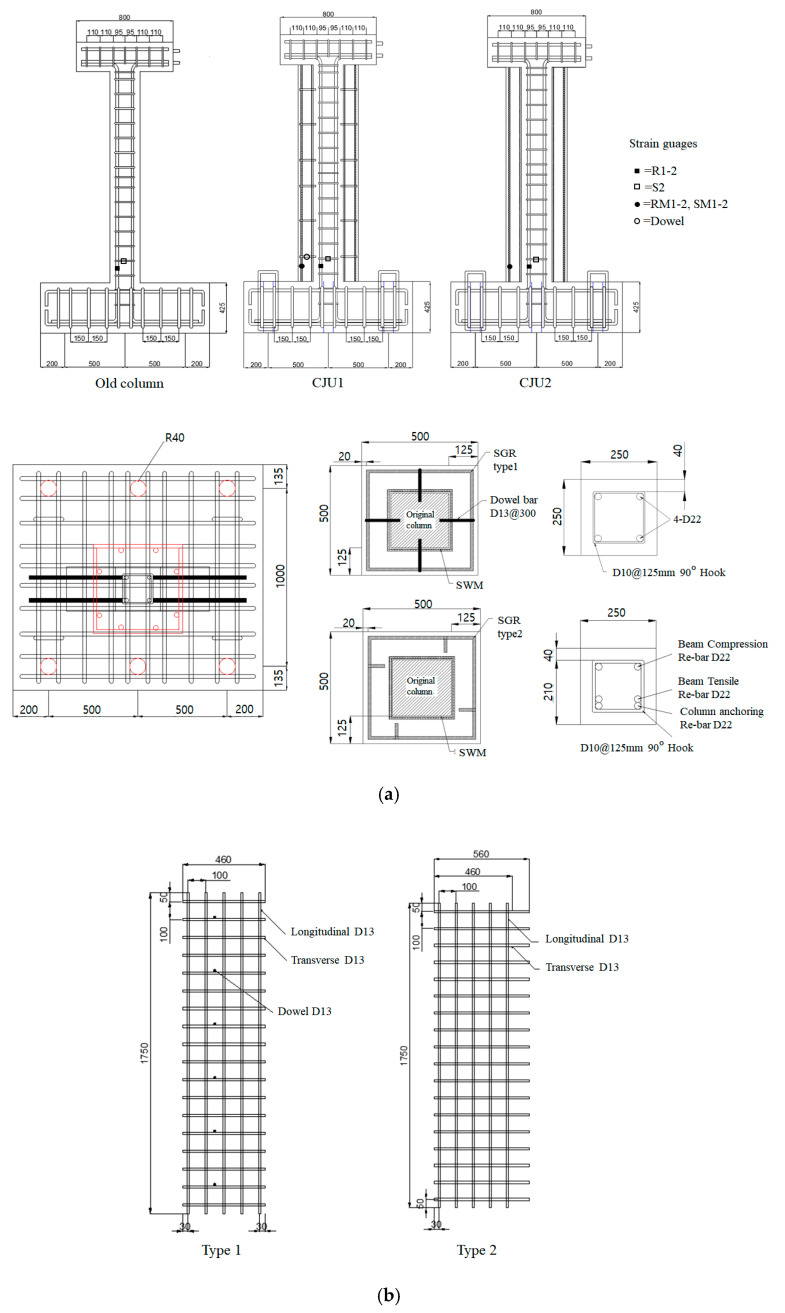
Details of the specimens (mm): (**a**) Sections of columns and footings; (**b**) the SGR of Type 1 and 2.

One non-retrofitted RC column specimen and two specimens retrofitted with each proposed detail were fabricated. The detailed drawings of the non-retrofitted RC specimen (CU) and two retrofitted specimens (CJU1, CJU2) are shown in Figure 2a, respectively. The details of the retrofit techniques and specimens are shown in Table 1 and Table 2, respectively. CJU1 and CJU2 are the names of specimens retrofitted with Type 1 and Type 2 reinforcement, respectively. The detailed drawings of each type of SGR are shown in Figure 2b. 

The old column was designed with a cross-sectional size of 250 mm × 250 mm in consideration of the minimum reinforcement spacing and concrete cover thickness specified in ACI 318-19 [8]. D22 longitudinal rebars were placed at the four corners. According to the Korean Design Standards 14 20 50 [9], the diameter and spacing of the stirrups were determined. D10 90-degree closed external stirrups were placed at spacings of 125 mm. The yield strengths of both the longitudinal rebars and external stirrups are 400 MPa. The height of the column is 1800 mm, and it was cast in the foundation of dimensions 1400 mm × 1270 mm × 450 mm. For fixing the foundation to the strong floor, bolt holes were drilled at spacings of 500 mm in the foundation.

**Figure 3:**
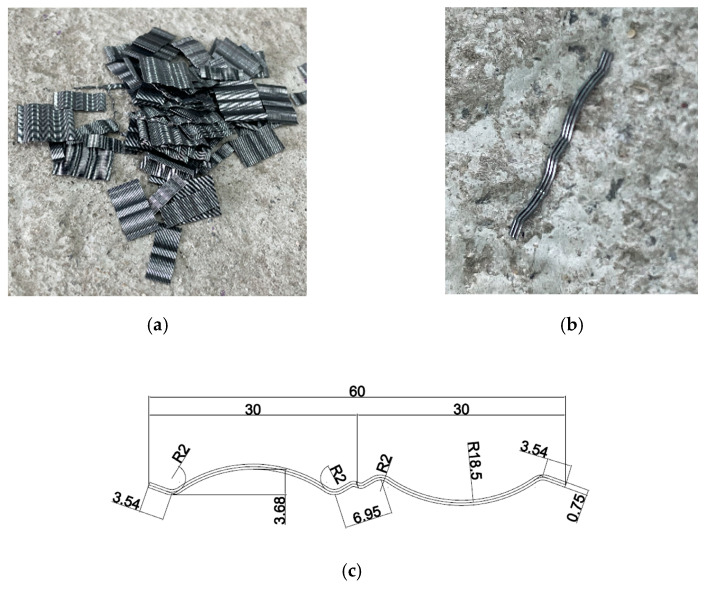
Steel fibers: (**a**) Before dispersion; (**b**) After dispersion; (**c**) Section details (mm).

The sizes of the jacketing section of the retrofitted specimens of CJU1 and CJU2 were each set to 500 mm × 500 mm in consideration of the minimum thickness for the dowel bar, minimum thickness for reinforcement, and concrete cover thickness. In the jacketed section of CJU1 and CJU2, steel fibers are mixed with 40.09 MPa non-shrinkage mortar. The steel fiber mixing ratio was set to 1.5%. The steel fiber mixed in SFNM is shown in Figure 3. It is roughened wire fiber, with a double-arched shape, and is glued in bundles. The fibers are filaments of wire, deformed, and cut to lengths, for the reinforcement of mortar focusing on crack resistance. The double-arched shaped steel fiber has a length of 18 mm and diameter of 0.34 mm, thus the aspect ratio is 0.019. The maximum tensile strength of the fiber is 1250 MPa. The properties of the steel fiber are shown in Table 3. SGR consists of a square opening with a narrow spacing of 100 mm × 100 mm for both the longitudinal and transverse rebars of D13, and spot welding was applied to each mesh. 

### 2.3. Test Setup and Loading Protocol

**Figure 4:**
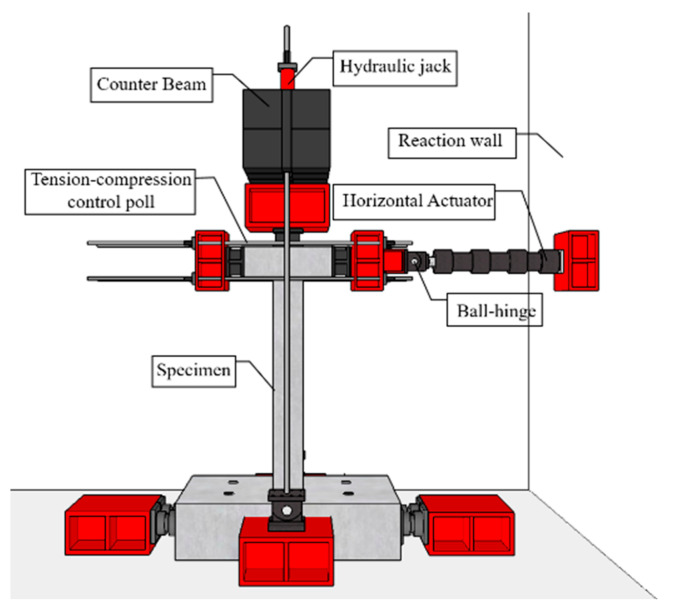
Test setup.

**Figure 5:**
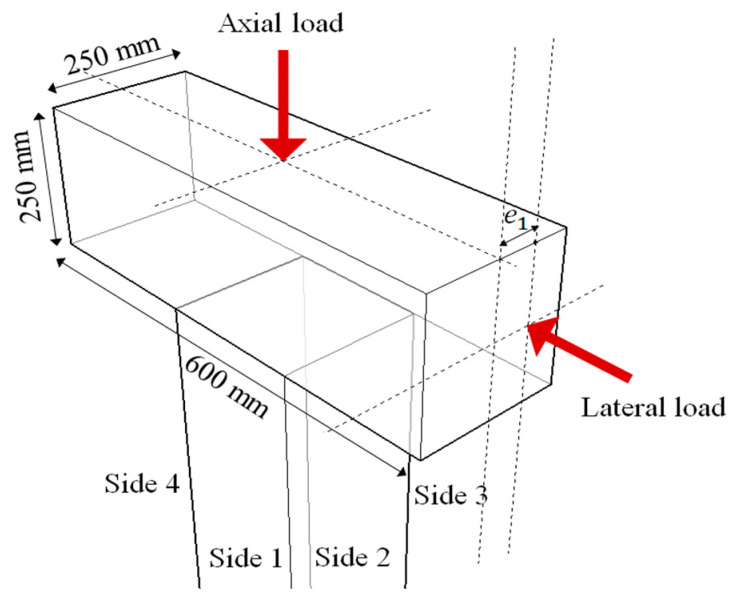
Loading method.

**Figure 6:**
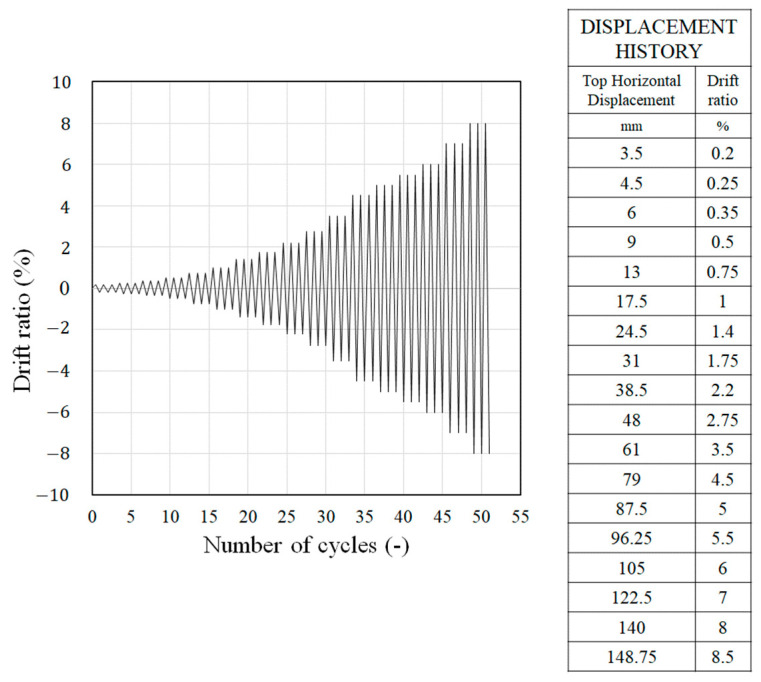
The loading protocol.

Three RC columns were tested to evaluate their behavior and capacity under combined cyclic load. Details of the loading setup are provided in Figure 4 and Figure 5. A jig was installed at the opposite end where the actuator was installed, and a repeated lateral load was applied by connecting both ends with a tension–compression control poll. At this time, the actuator was set with the eccentric distance in the horizontal direction on the horizontal center line of the upper beam such that the axis of axial load and lateral load did not coincide. When the axial load acts outside the core radius corresponding to one-sixth of the cross-sectional length, tensile force is generated. In this study, an eccentric distance ($e_{1}$) of 65 mm, which is one-fourth of the length of the cross-section, was applied to assume an extremely dangerous situation due to the generation of tensile force. An axial load was continuously applied to the upper beam by using a hydraulic. The constant axial load was 255 kN, which is 17% of the axial load capacity. The foundation was fixed to a strong floor, and steel rods were placed at spacings of 500 mm installed on the strong floor. To identify the direction, the front side of the specimen was named Side 1, and the elevations were divided by naming them Side 2, 3, and 4, in a counterclockwise direction. The lateral load was applied through a quasi-static protocol that is shown in Figure 6, according to the ACI 374.1-05 [10] standard. The lateral load was gradually increased from a drift ratio of 0.2%. The lateral displacement of the load point of all specimens was measured by a linear variable differential transducer (LVDTs). Two LVDTs were installed on each side of the top of the column to measure the displacement in the negative and positive directions. Strain gauges were attached at locations 150 mm from the bottom of the column to measure the strain of the longitudinal rebars, as shown in Figure 2a. Several sensors were attached at similar locations for data reliability. 

## 3. Experimental Results and Analysis

### 3.1. Observed Cracks and Failure Patterns

Table 4 shows the observed cracks and failure patterns at the end of the testing. In the CU specimen, three types of cracks occurred, including flexural cracks, shear cracks, and splitting cracks. The initial cracks were observed in the form of a flexural crack in the bottom of the column at a drift ratio of 1%. Shear cracks occurred and developed in the center of the column at a drift ratio of 2.2%. With the increase of loading, secondary cracks developed between the existing cracks and gradually progressed into shear cracks. Then, concrete spalling was observed as the shear crack widths increased. At a drift ratio of 2.75%, the load reached the maximum load of 21.6 kN and vertical splitting cracks were produced in the compression zone. Then, the vertical splitting cracks widened and extended upward. Severe cracks and concrete crushing occurred at the bottom of the column, which failed at a drift ratio of 4.5%.

CJU1 and CJU2 specimens retrofitted with the proposed concrete jacketing showed similar cracks and failure modes. At a drift ratio of 0.25%, flexural cracks occurred in the plastic hinge region. As the load increased, vertical splitting cracks were observed in the compression zone. Concrete spalling and crushing occurred at the bottom of the column in the CJU1 specimen at a drift ratio of 3.5% and in the CJU2 specimen at a drift ratio of 2.2%. Both specimens reached the maximum load at a drift ratio of 8%, and concrete crushing at the bottom of the column widened. Then, the load gradually decreased, and the experiment was terminated. 

In the CU specimen, more diagonal cracks appeared, and the angle of cracks became larger than for CJU1 and CJU2. This means that the failure modes of the CU specimens developed from flexural failure to torsional failure with the increase in torsional loading. Since the specimens retrofitted with a concrete jacket effectively resisted the torsional load, the specimens showed dominant flexural behavior. In addition, fewer cracks occur in the specimens retrofitted with concrete jacketing than in the CU specimen, which may be attributed to the contribution of the steel fiber to crack control. This is because the steel fiber acts as a bridge to mitigate an increase in crack width through pull-out resistance after cracks occur [11,12].

### 3.2. Load–Displacement Relationships

**Figure 7:**
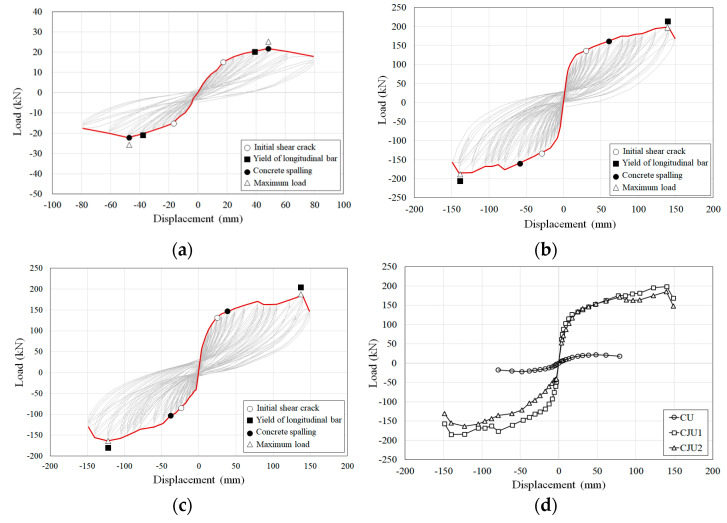
Hysteresis envelope curves: (**a**) CU; (**b**) CJU1; (**c**) CJU2; (**d**) Skeleton curves of all specimens.

The hysteresis curves and skeleton curves are shown in Figure 7. All specimens showed a linear elastic behavior before the development of flexural cracks. CU exhibited relatively narrow hysteresis loops compared to CJU1 and CJU2. In the case of CU, shear cracks occurred at 15 kN and longitudinal bars yielded at 18.3 kN. It was failed after reaching the maximum load of 21.6 kN. CJU1 and CJU2 showed significantly larger maximum loads comparing with CU. In the case of CJU1, longitudinal bars yielded, and maximum load was recorded at 198 kN. There are similar behavior patterns between CJU1 and CJU2. The longitudinal bars yielded, and maximum load was recorded at 185.2 kN in CJU2. In CU, the maximum load was reached after the yielding of the longitudinal bar, whereas in CJU1 and CJU2, the maximum load was recorded simultaneously with the yield of the longitudinal bar. In addition, CJU1 and CJU2 specimens reached the maximum load while resisting the load of more cycles after the concrete spalling. 

The maximum load and displacement for the positive and negative directions are shown in Table 5. The only difference between CJU1 and CJU2 is the yield and ultimate strength, but the difference was not significant. The maximum loads of CJU1 and CJU2 were 8.91 times and 8.32 times that of CU, respectively. In addition, the maximum displacement of each of these two proposed columns was 1.88 times that of CU, so it was confirmed that the retrofit technique effectively enhances the strength and deformation capacity of the column. On the other hand, the maximum load of CJU1 was about 1.1 times that of CJU2, confirming the similar performance of Type 1 and Type 2 in terms of strength improvement. Through this, both the dowel bar of Type 1 and the hook-shaped SGR coupling details for imposing additional confinement between the existing column and the jacketed section can produce a restraining effect, and it is considered possible that the SGR of Type 2 provides an appropriate confinement effect without additional dowel bar reinforcement. 

The predicted moment capacity *M_n_* of RC column is determined according to the ACI 318-19 [8]. The experimental value is the average of positive and negative moments.

(1) $$M_{n}=C_{c}\left(\frac{h}{2}-\frac{a}{2}\right)+\overset{n}{\underset{i=1}{\sum }}F_{si}\left(\frac{h}{2}-d_{i}\right)$$

(2) $$F_{si}=A_{si}\cdot f_{si}$$

where $C_{c}$ = the compressive force in the concrete; $A_{si}$ = the areas in each layer of longitudinal reinforcement; $d_{i}$ = the depth to ith layer of steel; $f_{si}$ = the stresses in each layer of reinforcement; $F_{si}$ = the forces in each layer of reinforcement; $\varepsilon_{si}$ = strain in the ith layer of reinforcement.

It is presented that the ACI equation overestimates the experimental value because the lateral force with eccentricity was applied, and the torsion occurred. However, both CJU1 and CJU2 showed moment more than four times greater than the design moment. This means that the proposed concrete jacket can significantly improve the performance of the existing RC columns. 

### 3.3. Stiffness Degradation

In this study, the stiffness of each specimen was evaluated by Equation (3), referring to Vandoros et al. [4].

(3) $$K_{i}=\left(\left|\frac{+F_{i}}{+\Delta_{i}}\right|+\left|\frac{-F_{i}}{-\Delta_{i}}\right|\right)/2$$

Here, $K_{i}$ means the stiffness at the *i*-th drift ratio. $\pm F_{i}$ means the maximum load in the positive and negative directions at the *i*-th drift ratio. $\pm \Delta_{i}$ means the maximum displacement in the positive and negative directions at the *i*-th drift ratio.

**Figure 8:**
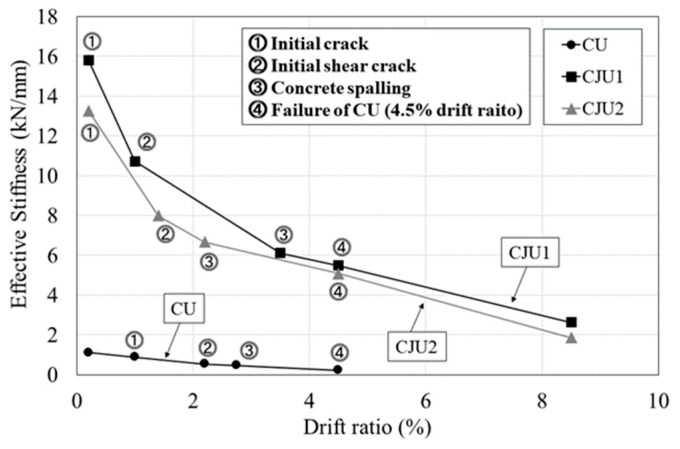
Comparison of the stiffness degradation of specimens.

All stiffnesses were calculated with the result of the first cycle of each drift ratio. The stiffness of each specimen and degradation patterns are shown in Table 6 and Figure 8, respectively. The points are divided by the occurrence of the initial crack, the occurrence of the shear crack, the occurrence of concrete spalling, and the maximum drift ratio of CU. The initial stiffness was 1.1 kN/mm, 15.81 kN/mm, and 13.27 kN/mm for CU, CJU1, and CJU2, respectively. The initial stiffnesses of CJU1 and CJU2 were about 12 times and 14 times higher than CU, respectively. At the drift ratio where the initial shear crack occurred, the stiffnesses of CU, CJU1, and CJU2 were 48%, 67%, and 60% of the initial stiffness, respectively. At the time of concrete spalling, the stiffnesses of CU, CJU1, and CJU2 were 42%, 52%, and 50% of the initial stiffnesses, respectively. The stiffness at the drift ratio of 4.5%, where the experiment on CU was terminated, is compared to the initial stiffness. It was confirmed that CU, CJU1, and CJU2 showed about 21%, 35%, and 38% of their initial stiffnesses, respectively. The stiffnesses of CJU1 and CJU2 increased about 16% and 15% on average compared to CU, respectively. In the case of CU, the stiffness degradation rates compared to the initial stiffnesses were about 20% on average, and those of CJU1 and CJU2 were both about 16%. The difference in the stiffness degradation rate of CU, CJU1, and CJU2 spanned a range of about 4%, and it was presented that the performance of reducing the stiffness degradation rate using the proposed detail did not significantly increase.

The proposed details increase the initial stiffness of reinforced concrete columns by about 13 times and effectively prevent the decrease in stiffness when cracks occur. Compared to CJU2, CJU1 showed a relatively small decrease in stiffness up to the time of concrete spalling, but it was confirmed that the difference in stiffness degradation between the two specimens decreased as the load increased. Until concrete spalling, the dowel bar of CJU1 secures the monolithic behavior of the existing column and jacketed section, and the hook-shaped SGR of CJU2 increases the restraining strength and reduces stiffness degradation.

### 3.4. Strains of Reinforcements

**Figure 9:**
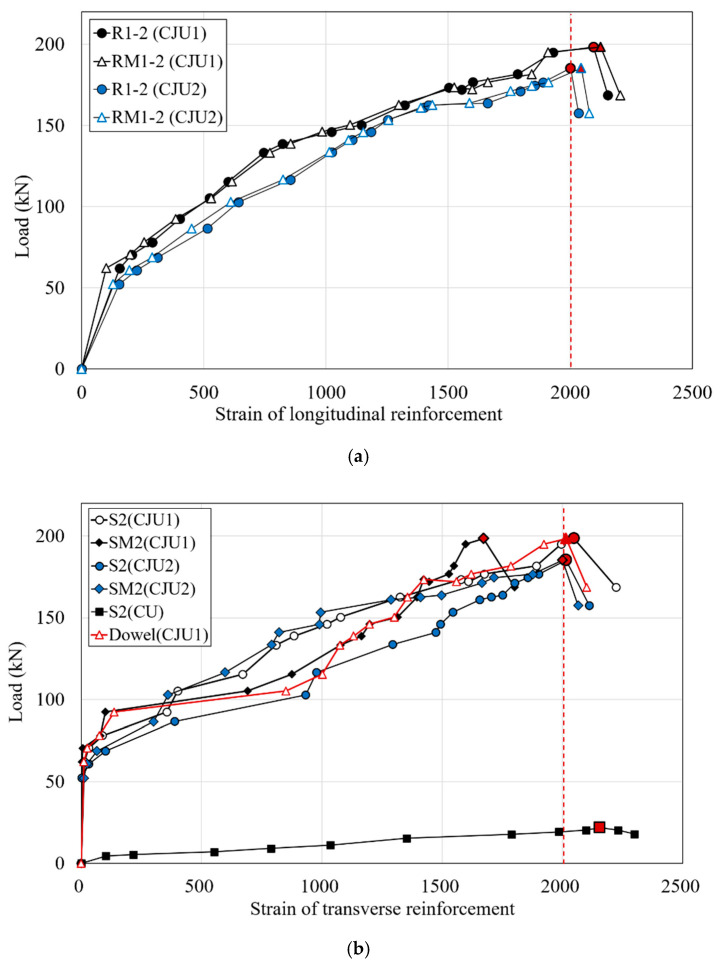
Strains of reinforcements: (**a**) longitudinal reinforcements; (**b**) transverse reinforcements.

In order to improve the effective performance of concrete jacketing, it is necessary to secure the bond and confinement between the existing member and the jacketed section. If the proper confinement and bond of the columns retrofitted by jacketing are not secured, the contact surface may be destroyed due to bond slip between the faces [13,14]. In this study, to evaluate the bond and confinement behavior of the concrete jacketed specimens, the strains of longitudinal rebars and stirrups are respectively shown in Figure 9. Strain gauges were attached at locations 150 mm from the bottom of the column to measure the strain of the longitudinal rebars. The R and S series of the strains are longitudinal rebars and stirrups of old columns, the RM and SM series are longitudinal and transverse bars of the SGR, respectively. The strain at the point of maximum load of each specimen was identified with a red mark. As shown in Figure 9a, the longitudinal rebars of the old column and longitudinal rebars of the SGRs of CJU1 and CJU2 yielded at the point of maximum load, and the strain increase before yield was similar. It is presented that the existing column and jacketed section behave monolithically until the maximum load is reached.

As shown in Figure 9b, in all specimens, a similar pattern was observed in which the strain of the stirrups of the old column increased as the applied load increased. Additionally, the strain of the transverse bars of the SGR gradually increased as the lateral load increased. However, in the jacketed section, a different pattern was observed for the strain of the transverse reinforcement of SGR in CJU1. In the case of CJU1, although the transverse bars of the SGR did not yield until the maximum load, it was confirmed that the dowel bar of CJU1 yielded after increasing strain until the maximum load. The dowel resist shear loads due to slip between the old column and the jacketed section. Therefore, the stress due to the shear load was concentrated on the dowel. Since the shear stress were well distributed by the dowel, the strain of the transverse reinforcement of SGR increased as the load increased. 

In both CJU1 and CJU2, the jacket reinforcement bars in SGR did not buckle before reaching the maximum load. This is because, without welding, the dowel bar or hooked SGR worked effectively and provided adequate confinement effect. In the case of CJU2, the coupling details of the Type 2 hook-shaped SGR exerted a confinement effect, considering that the transverse bars of SGR yielded at the maximum load. On the other hand, in the case of CU, the stirrups yielded before the point of maximum load, which is thought to be the result of the torsion occurring as the magnitude of the lateral load with eccentricity increased, and the external stirrups did not sufficiently resist torsion and shear.

### 3.5. Energy Dissipation

In this study, the energy dissipation capacity of each specimen was calculated by Equation (4) referring to Troung et al. [15], and it is the area of the first cycle of the bidirectional load–displacement curve.

(4) $$E_{D}=\sum F_{i}\Delta_{i}$$

Here, $E_{D}$ represents the energy dissipated for each cycle of the load–displacement hysteresis curve. $F_{i}$ is the average value of lateral loads of positive and negative direction of the *i*-th drift ratio. $\Delta_{i}$ is the average value of the displacements in the positive and negative direction of the *i*-th drift ratio. 

**Figure 10:**
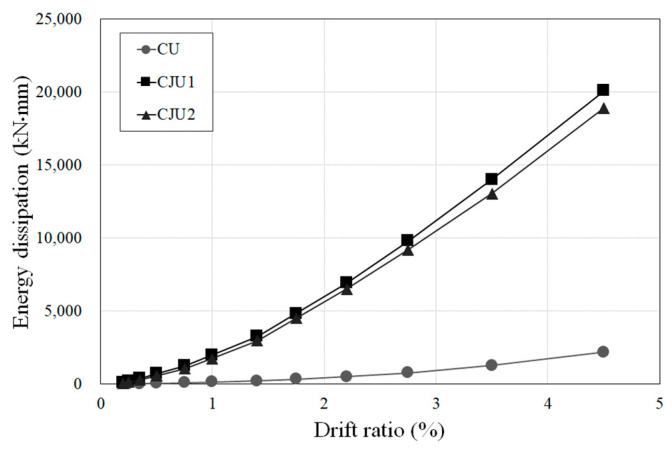
Cumulative dissipated energy for all specimens.

The increase in the energy dissipation capacity of each specimen according to the increase in the drift ratio is shown in Figure 10. All specimens showed a tendency to gradually increase their energy dissipation capacity as the drift ratio increased. The cumulative energy dissipation capacities up to the point of failure of CU, CJU1, and CJU2 were 2181.94 kN·mm, 69,263.57 kN·mm, and 47,091.86 kN·mm, respectively. The energy dissipation results of CJU1 and CJU2 below the drift ratio of 4.5% were 9.16 times and 8.67 times larger than that of CU, respectively. At the drift ratio of 4.5%, the energy dissipation capacity of CJU1 was about 1.06 times that of CJU2, and the cumulative energy dissipation capacity of CJU1 was about 1.4 times that of CJU2 at the drift ratio of 8.5%. As a result, there was no significant difference in energy dissipation capacity between CJU1 and CJU2.

## 4. Conclusions

In this study, two types of jacketing details that can improve both structural capacity and the constructability were proposed. In order to evaluate the structural performance of the RC columns retrofitted with the proposed concrete jacketing, cyclic loading tests were conducted in consideration of axial load, lateral load, and torsion. The following conclusions have been drawn:

(1) In the case of CU, it was confirmed that many shear cracks due to torsion were distributed. On the other hand, for CJU1 and CJU2, transverse cracks were evenly distributed along the column. Concrete cover spalling and crushing at the bottom of the column occurred somewhat, but the increases in the widths of the cracks were insignificant because of confinement effect provided by the proposed concrete jacket. Therefore, it has been demonstrated that proposed details, in combination with SGR, SWM, and SFNM, effectively resisted combined loading.
(2) The maximum load of CJU1 and CJU2 was about 8 times larger than that of CU and the initial stiffnesses of CJU1 and CJU2 were about 13 times larger than CU, respectively. It has been demonstrated that the strength and stiffness can be significantly improved by proposed concrete jacket details.
(3) The yield of the longitudinal reinforcement of the old column of the CJU1 and CJU2 was confirmed. Additionally, the separation of the jacket from the old column was not observed even if there was no surface treatment, and the role of the contact interface between the old column and new concrete layer was performed by SWM.
(4) RC columns retrofitted with the proposed details achieved significant increases in load carrying capacity, stiffness, and energy dissipation. The jacketed section with proposed details is constructed only by assembling the pre-fabricated reinforcement without surface treatment process. Simple details and easy installation can help practitioners to apply it easily. According to the current study, similar performance was exhibited regardless of the details. It is recommended to use concrete jacket details with hooked type without drilling and dowel fixing.

## Figures and Tables

**Table 1:** Comparison of reinforcement details between specimens.

Designation of Specimen	Retrofit Method	Component	Fixing
CU	-	-	-
CJU1	Type 1	SWM + SGR + Dowel bar	Dowel bar + Lap spliced
CJU2	Type 2	SWM + SGR	Hook

**Table 2:** Descriptions of the specimens.

Specimen	Cross-Section (mm)	Reinforcements		Materials	
		Longitudinal	Transverse	Concrete	Steel
CU	250 × 250	4-D22	D10@125	24 MPa	400 MPa
CJU1	500 × 500				
CJU2					

**Table 3:** The geometrical and mechanical properties of the steel fiber.

Material	Diameter (mm)	Length (mm)	Aspect Ratio	Tensile Strength (MPa)
Low carbon	0.34	18	0.0182	1250

**Table 4:** Crack patterns of specimens at failure.

Specimens	Side 1	Side 2	Side 3	Side 4
CU	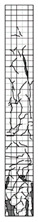	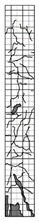	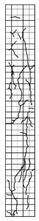	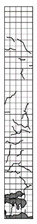
CJU1	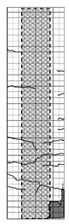	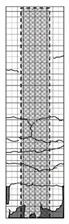	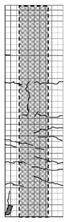	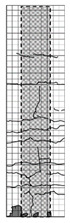
CJU2	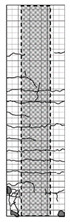	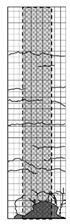	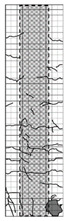	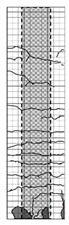

**Table 5:** Test results.

Specimen	ACI 318-19	Yield Point		Ultimate Point		Failure Point		$M_{test}$ (kNm)	$\bar{M_{test}}/M_{n}$
	$M_{n}$	$P_{y}$ (kN)	$\Delta_{y}$ (mm)	$P_{u}$ (kN)	$\Delta_{u}$ (mm)	$P_{f}$ (kN)	$\Delta_{f}$ (mm)		
CU	73.02	+18.56	38.56	+22.26	79.08	−22.26	79.08	39.49	0.54
CJU1		+198.32	140.08	+198.32	140.08	+168.57	148.76	344.77	4.72
CJU2		+185.23	139.49	+185.23	139.49	+130.51	148.76	313.90	4.30

+: *positive loading*. −: *negative loading*.

**Table 6:** The stiffnesses of specimens.

Specimen	Yield Point		Ultimate Point		Failure Point	
	$P_{y}$ (kN)	$\Delta_{y}$ (mm)	$P_{u}$ (kN)	$\Delta_{u}$ (mm)	$P_{f}$ (kN)	$\Delta_{f}$ (mm)
CU	38.56	20.29	22.26	79.08	−22.26	79.08
CJU1	198.32	140.08	198.32	140.08	168.57	148.76
CJU2	185.23	139.49	185.23	139.49	130.51	148.76

## Data Availability

Not applicable.

## References

[1] Julio E.S., Branco F., Silva V.D. (2005). Reinforced concrete jacketing-interface influence on monotonic loading response. ACI Struct. J..

[2] Julio E.S., Branco F., Silva V.D. (2008). Reinforced concrete jacketing-interface influence on cyclic loading response. ACI Struct. J..

[3] Raza S., Khan M.K., Menegon S.J., Tsang H.H., Wilson J.L. (2019). Strengthening and repair of reinforced concrete columns by jacketing: State-of-the-art review. Sustainability.

[4] Vandoros K.G., Dritsos S.E. (2008). Concrete jacket construction detail effectiveness when strengthening RC columns. Constr. Build. Mater..

[5] Sun Y., Zhang X. (2021). Study on the Seismic Performance of Strengthened Reinforced Concrete Columns Based on the Experiment. Geofluids.

[6] Tayeh B.A., Maraq M.A.A., Ziara M.M. (2020). Flexural performance of reinforced concrete beams strengthened with self-compacting concrete jacketing and steel welded wire mesh. Structures.

[7] Yang K.H., Kim W.W. (2016). Axial compression performance of reinforced concrete short columns with supplementary v-shaped ties. ACI Struct. J..

[8] (2019). Building Code Requirements for Structural Concrete and Commentary.

[9] (2021). Design Specification of Steel Reinforcement Details for Concrete Structure.

[10] (2005). Acceptance Criteria for Moment Frames Based on Structural Testing and Commentary.

[11] Gholampour A., Hassanli R., Mills J.E., Vincent T., Kunieda M. (2019). Experimental investigation of the performance of concrete columns strengthened with fiber reinforced concrete jacket. Constr. Build. Mater..

[12] Dadvar S.A., Mostofinejad D., Bahmani H. (2020). Strengthening of RC columns by ultra-high performance fiber reinforced concrete (UHPFRC) jacketing. Constr. Build. Mater..

[13] Suarjana M., Octora D.D., Riyansyah M. (2020). Seismic Performance of RC Hollow Rectangular Bridge Piers Retrofitted by Concrete Jacketing Considering the Initial Load and Interface Slip. J. Eng. Technol. Sci..

[14] Thermou G.E., Papanikolaou V.K., Kappos A.J. (2014). Flexural behaviour of reinforced concrete jacketed columns under reversed cyclic loading. Eng. Struct..

[15] Truong G.T., Kim J.C., Choi K.K. (2017). Seismic performance of reinforced concrete columns retrofitted by various techniques. Eng. Struct..

